# Comment on: Jilkova, Z.M.; et al. “Predictive Factors for Response to PD-1/PD-L1 Checkpoint Inhibition in the Field of Hepatocellular Carcinoma: Current Status and Challenges” *Cancers* 2019, *11*, 1554

**DOI:** 10.3390/cancers12092670

**Published:** 2020-09-18

**Authors:** Anna Dunnigan

**Affiliations:** Institute of Clinical Trials and Methodology, University College London, London WC1V 6LJ, UK; anna.dunnigan.19@ucl.ac.uk

I read with interest the article ‘Predictive Factors for Response to PD-1/PD-L1 Checkpoint Inhibition in the Field of Hepatocellular Carcinoma: Current Status and Challenges’ [1]. However, the authors’ assertion that ‘Certainly, high TMB and neoantigen load have been noted to predict the response to immunotherapies, including anti-PD-1 therapy (higher objective response rate and/or prolonged survival) in melanoma, non-small-cell lung carcinoma’ is not supported by the reference provided—an article by Topalian et al. [2]—which mentions neither tumour mutational burden (TMB) nor neoantigen load. A better reference might have been a retrospective review published by Goodman et al. in 2017 [3] in which 151 immunotherapy-treated patients who had undergone TMB assessment were analysed, and a linear correlation between higher TMB and better outcome parameters was identified. Nonetheless, I would also suggest that a definitive conclusion should not be based purely on a retrospective review in any case, and that a prospective study or clinical trial is needed to support inferences about the ability of TMB to predict response to PD-1 inhibitors.

## References

[1] Macek Jilkova Z., Aspord C., Decaens T. (2019). Predictive Factors for Response to PD-1/PD-L1 Checkpoint Inhibition in the Field of Hepatocellular Carcinoma: Current Status and Challenges. Cancers.

[2] Topalian S.L., Hodi F.S., Brahmer J.R., Gettinger S.N., Smith D.C., McDermott D.F., Powderly J.D., Carvajal R.D., Sosman J.A., Atkins M.B. (2012). Safety, activity, and immune correlates of anti-PD-1 antibody in cancer. N. Engl. J. Med..

[3] Goodman A.M., Kato S., Bazhenova L., Patel S.P., Frampton G.M., Miller V., Stephens P.J., Daniels G.A., Kurzrock R. (2017). Tumor Mutational Burden as an Independent Predictor of Response to Immunotherapy in Diverse Cancers. Mol. Cancer Ther..

